# Synergistic interaction of high blood pressure and cerebral beta-amyloid on tau pathology

**DOI:** 10.1186/s13195-022-01149-7

**Published:** 2022-12-24

**Authors:** Taewon Kim, Dahyun Yi, Min Soo Byun, Hyejin Ahn, Joon Hyung Jung, Nayeong Kong, Min Jung Kim, Gijung Jung, Jun-Young Lee, Yun-Sang Lee, Yu Kyeong Kim, Dong Young Lee, for the KBASE Research Group

**Affiliations:** 1 Department of Neuropsychiatry, Seoul National University Hospital, Seoul, Republic of Korea; 2 Institute of Human Behavioral Medicine, Medical Research Center Seoul National University, Seoul, Republic of Korea; 3 Department of Psychiatry, Seoul National University College of Medicine, Seoul, Republic of Korea; 4 Department of Neuropsychiatry, Nowon Eulji University Hospital, Seoul, Republic of Korea; 5 Department of Neuropsychiatry, Seoul Metropolitan Government-Seoul National University Boramae Medical Center, Seoul, Republic of Korea; 6 Department of Nuclear Medicine, Seoul National University College of Medicine, Seoul, Republic of Korea; 7 Department of Nuclear Medicine, Seoul Metropolitan Government-Seoul National University Boramae Medical Center, Seoul, Republic of Korea

**Keywords:** Hypertension, Blood pressure, Alzheimer’s disease, Positron emission tomography, Beta-amyloid, Tau

## Abstract

**Background:**

Hypertension has been associated with Alzheimer’s disease (AD) dementia as well as vascular dementia. However, the underlying neuropathological changes that link hypertension to AD remain poorly understood. In our study, we examined the relationships of a history of hypertension and high current blood pressure (BP) with in vivo AD pathologies including β-amyloid (Aβ) and tau and also investigated whether a history of hypertension and current BP respectively affect the association between Aβ and tau deposition.

**Methods:**

This cross-sectional study was conducted as part of the Korean Brain Aging Study for Early Diagnosis and Prediction of Alzheimer’s Disease, a prospective cohort study. Cognitively normal older adults who underwent both Aβ and tau positron emission tomography (PET) (i.e., [^11^C]-Pittsburgh compound B and [^18^F] AV-1451 PET) were selected. History of hypertension and current BP were evaluated and cerebral Aβ and tau deposition measured by PET were used as main outcomes. Generalized linear regression models were used to estimate associations.

**Results:**

A total of 68 cognitively normal older adults (mean [SD] age, 71.5 [7.4] years; 40 women [59%]) were included in the study. Neither a history of hypertension nor the current BP exhibited a direct association with Aβ or tau deposition. However, the synergistic interaction effects of high current systolic (β, 0.359; SE, 0.141; *p* = 0.014) and diastolic (β, 0.696; SE, 0.158; *p* < 0.001) BP state with Aβ deposition on tau deposition were significant, whereas there was no such effect for a history of hypertension (β, 0.186; SE, 0.152; *p* = 0.224).

**Conclusions:**

The findings suggest that high current BP, but not a history of hypertension, synergistically modulate the relationship between cerebral Aβ and tau deposition in late-life. In terms of AD prevention, the results support the importance of strict BP control in cognitively normal older adults with hypertension.

**Supplementary Information:**

The online version contains supplementary material available at 10.1186/s13195-022-01149-7.

## Background

Hypertension is related to a higher risk of dementia and more rapid cognitive decline in older adults [1–5]. More specifically, hypertension is associated not only with the development of vascular dementia and vascular cognitive impairment [6–8], but also with that of Alzheimer’s disease (AD) dementia [9–11]. However, the underlying pathological changes that link hypertension to AD remain poorly understood.

While one postmortem study showed that midlife hypertension was associated with more senile plaque and neurofibrillary tangle pathologies at death [12], other studies reported that late-life high blood pressure (BP) was related only to neurofibrillary tangles or even to neither neurofibrillary tangles nor senile plaques [13, 14]. In vivo AD biomarker studies based on positron emission tomography (PET) imaging or cerebrospinal fluid (CSF) analysis yielded similarly conflicting findings. Although a couple of studies demonstrated a significant association between high BP and cerebral β-amyloid (Aβ) deposition in older adults [15, 16], many other studies did not find such direct relationship [17–24]. In terms of tau pathology, while a recent CSF study revealed that current BP had a significant positive association with tau level [17] and a PET study showed that systolic BP (SBP) synergistically interacted with Aβ on tau deposition [25], other studies reported that a history of hypertension was not related to CSF tau level or tau deposition on PET [19, 24]. As the current BP level reflects the appropriateness of hypertension management and a large proportion of hypertensive individuals exhibit poorly controlled BP, current BP status may explain the adverse effects of hypertension better than a history thereof [26–28]. However, few studies have evaluated the effects of both a history of hypertension and current BP on AD pathologies in the same population.

In this context, we aimed to examine the relationships of a history of hypertension and current BP with in vivo AD pathologies (cerebral Aβ and tau deposition on PET) in cognitively normal (CN) older adults. We also investigated the modulatory effects of hypertension and current BP on the association between Aβ and tau deposition.

## Methods

### Participants

This study was performed as part of the Korean Brain Aging Study for the Early Diagnosis and Prediction of Alzheimer’s Disease (KBASE), an ongoing prospective cohort study commenced in 2014. The KBASE study aimed to search for new AD biomarkers and investigate how different lifetime experiences and bodily changes contribute to the brain alterations related to AD [29]. The subject recruitment process was described previously [29]. Among the overall subjects of the KBASE cohort, 68 CN older adults (aged 55–90 years) who underwent both amyloid and tau PET imaging were included in the present study. All CN participants were free from diagnosis of mild cognitive impairment or dementia; they all had global Clinical Dementia Rating score of 0. The exclusion criteria were any serious medical, psychiatric, or neurological disorders that could affect mental functioning; history of loss of consciousness after head trauma; severe communication or behavioral problems that would make clinical examination or brain scans difficult; illiteracy; any significant visual or hearing difficulty; and participation in clinical trial with an investigational product. The study protocol was approved by the Institutional Review Boards of Seoul National University Hospital and Seoul Metropolitan Government-Seoul National University Boramae Medical Center, Seoul, South Korea. The study was performed in accordance with the recommendations of the current version of the Declaration of Helsinki. All participants provided written informed consent.

### Clinical assessment

All participants were examined by neuropsychiatrists with advanced training in dementia research according to the KBASE clinical assessment protocol, which incorporates the Korean version of the Consortium to Establish a Registry for Alzheimer’s Disease (CERAD) Assessment Packet [30]. A history of hypertension was defined as either a documented diagnosis of hypertension or treatment with antihypertensive medication, based on data collected by trained research nurses via interviews with participants and reliable informants. SBP and diastolic BP (DBP) were manually measured three times by a trained nurse at 5-min intervals in supine position after 5 min of rest. The mean SBP and DBP were used for the analysis. SBP and DBP were also categorized into high or low state using the following cutoffs: ≥ 140 vs. < 140 mmHg, and ≥ 90 vs. < 90 mmHg, respectively [31]. Comorbid vascular risk factors other than hypertension (diabetes mellitus, hyperlipidemia, coronary artery disease, transient ischemic attack, and stroke) were assessed in the same manner as the evaluation of hypertension history. A vascular risk score (VRS) reflecting the vascular burden other than hypertension was calculated based on the number of vascular risk factors other than hypertension [32].

### Measurement of cerebral Aβ and tau deposition

All participants underwent three-dimensional [^11^C]-Pittsburgh compound B (PiB)-PET using a 3.0 T Biograph mMR scanner (Siemens, Washington DC, USA) at the visit for clinical assessment including BP measurement. The details of PiB-PET image acquisition and preprocessing were described in our previous report [33]. The autonomic anatomical labeling algorithm and region combining method were used to set the regions of interest (ROIs) when characterizing the PiB retention in the frontal, lateral parietal, posterior cingulate-precuneus, and lateral temporal cortices [34, 35]. The standardized uptake value ratio (SUVR) for each ROI was calculated by dividing each value by the mean cerebellar uptake value in the same image. A global cortical ROI comprising the four ROIs was also defined, and a global Aβ retention value was generated by dividing the mean value for all voxels of the global cortical ROI by the mean cerebellar uptake value in the same image.

Participants also underwent [^18^F] AV-1451 PET scans using a Biograph True point 40 PET/CT scanner (Siemens, USA) per the manufacturer’s approved guidelines. While PiB-PET was performed during the baseline visit, AV-1451 PET imaging was performed at an average of 72 days after the baseline visit. The details of AV-1451 PET image acquisition and preprocessing were described in our previous report [33]. AV-1451 PET SUVR images based on the mean uptake over 80 to 100 min post-injection were normalized by the mean inferior cerebellar gray matter uptake, according to the published code [36]. To estimate cerebral tau deposition, we quantified the partial volume-corrected AV-1451 SUVR of inferior temporal (IT) ROI, which is the neocortical site of tau deposition in early AD [37, 38].

### Statistical analysis

For demographic and clinical characteristics, comparisons between participants with and without a history of hypertension were performed using the *χ*^2^ test or Fisher’s exact test for categorical data, and the independent *t*-test for continuous data. We used generalized linear regression model (GLM) to determine whether global Aβ and IT tau deposition were directly associated with a history of hypertension and high current BP (SBP and DBP; using both categorical and continuous variable) after adjusting for age, sex, apolipoprotein ε4 (APOE4) positivity, and the VRS. To analyze the moderating effect of a history of hypertension and current BP on the association between Aβ on tau deposition, a similar GLM including an interaction term of history of hypertension (or current SBP or DBP) × global Aβ retention as an additional independent variable was used. All statistical analyses utilized SPSS software (version 22.0, SPSS Inc; Chicago, IL, USA), and two-tailed *p-*values < 0.05 were considered statistically significant.

## Results

### Participant characteristics

A total of 68 participants (mean [SD] age, 71.5 [7.4] years; 40 women [59%]) were included in the study. Their demographic and clinical characteristics are summarized in Table 1. Of the 34 participants with a history of hypertension, 32 (94%) were on antihypertensive medication. Participants with a history of hypertension had higher VRS than the others, but there was no difference in age, sex, education, APOE4 positivity, SBP, or DBP between the two groups.

**Table 1:** Demographic and clinical characteristics of participants

Characteristics	History of hypertension (*n* = 34)	No history of hypertension (*n* = 34)	*p*-value
Age in years, mean (SD)	72.2 (8.0)	70.7 (6.7)	0.425
Females, (%)	21 (62)	19 (56)	0.622
Education in years, mean (SD)	12.1 (4.2)	11.9 (4.0)	0.906
APOE4 carrier, (%)	7 (21)	5 (15)	0.525
SBP in mmHg, mean (SD)	128.8 (14.9)	124.3 (19.4)	0.281
DBP in mmHg, mean (SD)	78.1 (10.2)	75.0 (11.9)	0.253
Onset age of hypertension in years, mean (SD)	60.6 (8.6)	-	
Duration of hypertension in years, mean (SD)	11.6 (7.0)	-	
Treatment with antihypertensive medication, (%)	32 (94)	-	
Diabetes mellitus, (%)	12 (35)	8 (24)	0.287
Coronary artery disease^a^, (%)	4 (12)	1 (3)	0.356
Dyslipidemia, (%)	17 (50)	12 (35)	0.220
Stroke, (%)	1 (3)	0	
TIA	0	0	
VRS, mean (SD)	1.00 (0.78)	0.62 (0.78)	0.047
Global WMH volume in mL, mean (SD)	17.53 (15.04)	12.00 (13.00)	0.108

*Abbreviations*: *APOE4* apolipoprotein E ε4, *SBP* systolic blood pressure, *DBP* diastolic blood pressure, *TIA* transient ischemic attack, *VRS* vascular risk score, *WMH* white matter hyperintensity

^a^ Fisher’s exact test was used for data comparison; other categorical characteristics were compared using the chi-square test and the continuous characteristics were compared using the independent *t*-test

### Relationships of a history of hypertension and current BP with in vivo AD pathologies

**Fig. 1:**
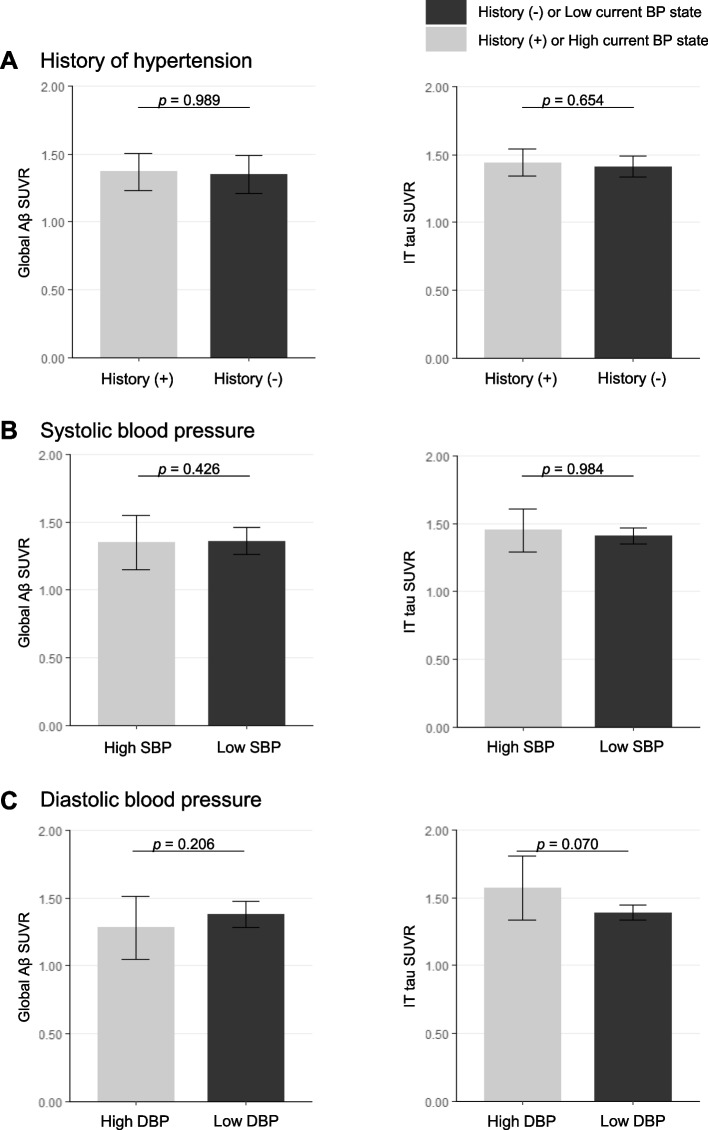
Global Aβ and IT tau deposition according to **A** history of hypertension, **B** current SBP state, and **C** current DBP state. The *p*-values are from linear regression models adjusted for age, sex, apolipoprotein E ε4 positivity, and the vascular risk score. Abbreviations: Aβ, β-amyloid; IT, inferior temporal; SBP, systolic blood pressure; DBP, diastolic blood pressure; BP, blood pressure; SUVR, standardized uptake value ratio

Neither global Aβ nor IT tau deposition was significantly different according to history of hypertension and high current SBP or DBP state (categorical variable), although IT tau deposition was marginally greater in high- than low-DBP group (Tables 2 and 3; Fig. 1). Current SBP or DBP (continuous variable) also exhibited no significant associations with Aβ and tau deposition (Table 4).

**Table 2:** Cerebral Aβ and tau deposition in participants with and without a history of hypertension

Variables	History of hypertension (*n* = 34)	No history of hypertension (*n* = 34)	Regression results^a^	
			β (SE)	*p*-value
Global Aβ deposition, mean (SD)	1.367 (0.400)	1.354 (0.407)	−0.001 (0.094)	0.989
IT tau deposition, mean (SD)	1.438 (0.308)	1.411 (0.238)	0.030 (0.068)	0.654

*Abbreviations*: *Aβ* β-amyloid, *IT* inferior temporal, *SE* standard error

^a^ Each linear regression model for global Aβ or IT tau deposition included a history of hypertension as an independent variable, after adjusting for age, sex, apolipoprotein E ε4 positivity, and the vascular risk score

**Table 3:** Cerebral Aβ and tau deposition in participants with and without high current blood pressure

Variables	High current SBP (*n* = 22)	Low current SBP (*n* = 46)	Regression results^a^	
			β (SE)	*p*-value
Global Aβ deposition, mean (SD)	1.354 (0.483)	1.364 (0.360)	−0.079 (0.099)	0.426
IT tau deposition, mean (SD)	1.449 (0.377)	1.413 (0.210)	0.001 (0.072)	0.984
Variables	High current DBP (*n* = 12)	Low current DBP (*n* = 56)	Regression results^a^	
			β (SE)	*p*-value
Global Aβ deposition, mean (SD)	1.354 (0.483)	1.364 (0.360)	−0.153 (0.120)	0.206
IT tau deposition, mean (SD)	1.449 (0.377)	1.413 (0.210)	0.158 (0.085)	0.070

*Abbreviations*: *Aβ* β-amyloid, *IT* inferior temporal, *SBP* systolic blood pressure, *DBP* diastolic blood pressure, *SE* standard error

^a^ Each linear regression model for global Aβ or IT tau deposition included high current SBP (≥ 140 mmHg) or DBP state (≥ 90 mmHg) as a categorical independent variable, after adjusting for age, sex, apolipoprotein E ε4 positivity, and the vascular risk score

**Table 4:** Associations of current systolic and diastolic blood pressure level with cerebral Aβ and tau deposition

Variables^a^	β (SE)	*p*-value
SBP (continuous)		
Global Aβ	−0.003 (0.003)	0.280
IT tau	0.000 (0.002)	0.973
DBP (continuous)		
Global Aβ	−0.008 (0.004)	0.082
IT tau	0.002 (0.003)	0.511

*Abbreviations*: *Aβ* β-amyloid, *SBP* systolic blood pressure, *IT* inferior temporal, *DBP* diastolic blood pressure, *SE* standard error

^a^ Each linear regression model for global Aβ or IT tau deposition included SBP or DBP level as a continuous independent variable, after adjusting for age, sex, apolipoprotein E ε4 positivity, and the vascular risk score

### Interaction effect between a history of hypertension (or current BP) and Aβ deposition on tau deposition

**Fig. 2:**
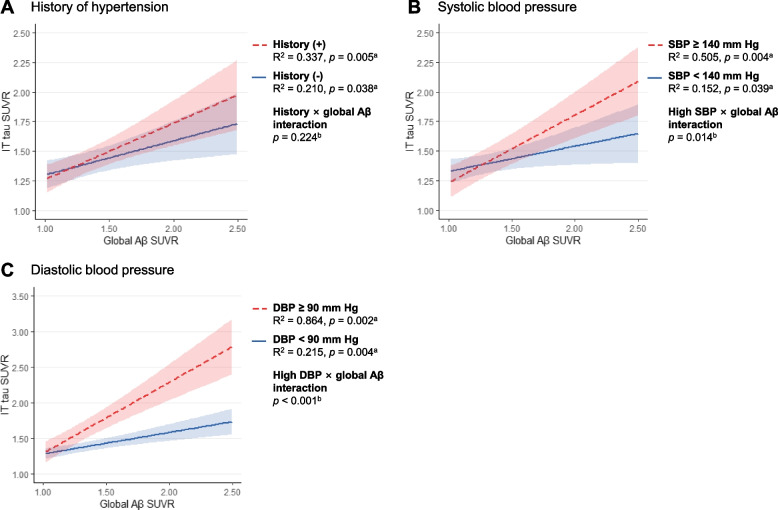
Moderating effects of **A** history of hypertension, **B** current SBP state, and **C** current DBP state on the relationship between global Aβ and IT tau deposition. ^a^ Each line and statistical values represent the relationship between cerebral Aβ and tau stratified by each blood pressure marker (e.g., history of hypertension, current SBP state, or current DBP state), based on the repeated linear regression model for tau deposition using global Aβ SUVR as independent variable with adjusting for age, sex, apolipoprotein E ε4 positivity, and the vascular risk score. Shaded regions demonstrate the 95% confidence interval for each regression line. ^b^ The *p*-values are from the generalized regression models including interaction term of each blood pressure marker and global Aβ SUVR, as well as each blood pressure marker, global Aβ SUVR, age, sex, apolipoprotein E ε4 positivity, and the vascular risk score. Abbreviations: SBP, systolic blood pressure; DBP, diastolic blood pressure; Aβ, β-amyloid; IT, inferior temporal; SUVR, standardized uptake value ratio

The interaction effect of a history of hypertension with global Aβ deposition on IT tau deposition was not significant (β, 0.186; SE, 0.152; *p* = 0.224) (Table 5 and Fig. 2A). In contrast, significant synergistic interaction effects of high current SBP and DBP states (categorical variables) with global Aβ on IT tau deposition were observed (β, 0.359; SE, 0.141; *p* = 0.014; β, 0.696; SE, 0.158; *p* < 0.001, respectively) (Table 5, Fig. 2B and C). Similarly, current SBP and DBP (continuous variables) also showed significant synergistic interactions (β, 0.011; SE, 0.004; *p* = 0.005; β, 0.019; SE, 0.006; *p* = 0.002, respectively) with global Aβ deposition on IT tau deposition (Table 5). As shown in Fig. 2B and C, the relationship between Aβ and tau deposition was stronger in individuals with high SBP (or DBP) than those with low SBP (or DBP).

**Table 5:** Interaction effects of history of hypertension or current blood pressure with global Aβ on inferior temporal tau deposition

	β (SE)	*p*-value
IT tau ~ BP marker × global Aβ + BP marker + global Aβ + age + sex + APOE4 + VRS^a^		
History of hypertension × global Aβ	0.186 (0.152)	0.224
High current SBP state × global Aβ	0.359 (0.141)	0.014
High current DBP state × global Aβ	0.696 (0.158)	< 0.001
Current SBP (continuous) × global Aβ	0.011 (0.004)	0.005
Current DBP (continuous) × global Aβ	0.019 (0.006)	0.002

*Abbreviations*: *Aβ* β-amyloid, *BP* blood pressure, *APOE4* apolipoprotein E ε4 positivity, *VRS* vascular risk score, *SBP* systolic blood pressure, *DBP* diastolic blood pressure, *SE* standard error

^a^ Summary of generalized linear regression model, repeated for each blood pressure marker (e.g., history of hypertension, high current blood pressure state, or current blood pressure level) and global Aβ as interactive predictors of tau deposition

## Discussion

In the current study, neither a history of hypertension nor the current BP was directly associated with Aβ or tau deposition (Tables 2 and 3; Fig. 1). However, the synergistic interaction effect of current BP with Aβ on tau deposition was significant, whereas the interaction effect between a history of hypertension and Aβ was not (Table 5 and Fig. 2). Our finding of no direct association of hypertension history or current BP with Aβ deposition is in line with many previous in vivo AD biomarker studies [17–24], although one postmortem study reported a positive association between BP measured in midlife and senile plaque pathology at death [12] and a biomarker study reported a positive relationship between BP and Aβ deposition in late middle-aged adults [15]. Taken together, although we cannot exclude the possibility that high BP in midlife may influence cerebral Aβ accumulation, high current BP or history of hypertension in late-life does not seem to be directly related to an increased Aβ burden.

We found no significant direct association of current BP or hypertension history with cortical tau deposition as well, which is comparable to the results in previous CSF and PET studies [18, 19, 24]. In contrast to these findings, a recent Chinese study reported a significant association of hypertension history and higher SBP with greater CSF tau level in individuals aged between 40 and 90 years [17]. However, its subgroup analyses revealed that association was significant only for the younger subgroup (< 65 years of age), but not for the older subgroup (≥ 65 years of age), of which the age distribution and result are similar to those of our study.

While we found no direct association between current BP and tau deposition, there was a significant synergistic interaction effect of current SBP and DBP with Aβ on tau deposition. The relationship between Aβ and tau deposition was greater in individuals with high SBP (or DBP) than in those with low SBP (or DBP), suggesting that current (late-life) BP moderates the relationship between cerebral Aβ and tau. Similarly, a synergistic interaction of SBP and cerebral Aβ burden on cortical tau deposition was recently reported, although the effect of DBP was not investigated [25]. It is difficult to determine the exact mechanism by which high BP acts in AD pathobiology. However, decreased cerebral blood flow and damaged blood-brain barrier, both of which can be caused by high BP, might be the contributing factors since both of them interact with Aβ to increase tau deposition [39]. Tau pathology may be exacerbated by increased tau hyperphosphorylation associated with chronic cerebral hypoperfusion and reduced tau clearance related with impairment of blood-brain barrier and glymphatic system [40–42]. Additionally, given that tau pathology is related to the missorting of axonal tau into somatodendritic compartment by Aβ deposition and other neuronal insults [43], we assessed the potential mediation of cerebrovascular insult, measured by white matter hyperintensities (WMHs), for the synergistic interaction effect of current BP with Aβ on tau deposition. We first analyzed the interaction effect of global WMH volume (instead of a history of hypertension or current BP) with global Aβ on tau deposition. As shown in Additional file 1: Supplementary Table 1, the interaction effect was not significant. Furthermore, the results presented in Table 5 were largely unchanged even when we controlled global WMH volume as an additional covariate (See Additional file 1: Supplementary Table 2). These findings indicate that cerebrovascular injury measured by WMHs does not mediate the synergistic effect of high BP on tau pathology.

In contrast to high current BP, the interaction effect of a history of hypertension with Aβ on tau deposition was not significant. This may support the importance of strict BP monitoring and control in individuals with hypertension in order to prevent AD-related cognitive decline. Multiple studies reported that individuals with untreated hypertension or less intensive BP control were at higher risk of cognitive decline [44–46]. In the present study, most (32/34, 94%) participants with a history of hypertension were on antihypertensive medication. However, 14 (44%) of them still had high current SBP or DBP, i.e., poorly controlled hypertension.

A novel point of this study is that we specifically identified that high SBP and DBP in late-life, but not a history of hypertension, synergistically interact with in vivo cerebral amyloid deposition on tau accumulation. However, several limitations should be considered. First, as the study was cross-sectional, we could not infer causality in the relationship between high BP and AD pathology. In addition, long-term BP variability may be a more important factor than BP at single point [47]. Further longitudinal studies with larger sample size and sufficient long-term BP variability data are required. Second, the participants had relatively narrow ranges of BP (SBP, 80 mmHg to 160 mmHg; DBP, 50 mmHg to 100 mmHg). Even in the group with a history of hypertension, most individuals (32/34, 94%) were on anti-hypertensive medication and only a few had very high BP (three with SBP ≥ 160 mmHg and three with DBP ≥ 100 mmHg). This might have reduced the likelihood of detecting any direct association between very high BP and in vivo AD pathology. Third, we did not consider the effect of specific details for antihypertensive medication history, such as onset, duration, and dosage of the medication, and specific drug(s) administered, although such details may affect the association between hypertension history and AD biomarkers. Finally, the impact of cerebral blood flow alteration, caused by hypertension, on PET radioligand binding dynamics needs to be considered when interpreting the results. High BP reduces cerebral blood flow, which can potentially decrease estimates of PET ligand binding (i.e., estimates of Aβ or tau deposition) in the brain [48, 49]. However, given that high BP, synergistically with Aβ, increased cerebral tau deposition rather than decreased it, it is not likely that the influence of cerebral blood flow on estimates of PET ligand binding was a major determinant of our results.

## Conclusion

Our findings suggest that high current BP, rather than a history of hypertension, may synergistically modulate the relationship between cerebral Aβ burden and tau deposition in later life. In terms of AD prevention, our results support the importance of strict BP control in cognitively normal older adults with hypertension.

## Supplementary Information

**Additional file 1: Supplementary Table 1.** Interaction effect of global WMH volume and global Aβ on inferior tau deposition. **Supplementary Table 2.** Interaction effect of BP marker and global Aβ on inferior temporal tau deposition with additional adjustment with global WMH volume.

## Data Availability

The data of the current research are not freely accessible because the Institutional Review Boards of Seoul National University Hospital prohibits public data-sharing for privacy reasons. However, the data may be available from the independent data-sharing committee of the KBASE research group on reasonable request, after approval by the Institutional Review Boards. Requests for data access can be submitted to the administrative coordinator of the KBASE group by e-mail (kbasecohort@gmail.com); the coordinator is independent of the authors.

## Abbreviations

AD: Alzheimer’s disease
BP: Blood pressure
Aβ: β-amyloid
PET: Positron emission tomography
CSF: Cerebrospinal fluid
SBP: Systolic blood pressure
CN: Cognitively normal
KBASE: Korean Brain Aging Study for the Early Diagnosis and Prediction of Alzheimer’s Disease
CERAD: Consortium to Establish a Registry for Alzheimer’s Disease
DBP: Diastolic blood pressure
VRS: Vascular risk score
PiB: Pittsburgh compound B
ROI: Region of interest
SUVR: Standardized uptake value ratio
IT: Inferior temporal
GLM: Generalized linear regression model
APOE4: Apolipoprotein ε4
WMH: White matter hyperintensity
