# Supplementary material for: Synergistic interaction of high blood pressure and cerebral beta-amyloid on tau pathology
Source: Alzheimers Res Ther. 2022 Dec 24;14:193. doi: 10.1186/s13195-022-01149-7 (PMC9789538; doi:10.1186/s13195-022-01149-7)
Supplement: Supplementary file 1 — Additional file 1: Supplementary Table 1. Interaction effect of global WMH volume and global Aβ on inferior tau deposition. Supplementary Table 2. Interaction effect of BP marker and global Aβ on inferior temporal tau deposition with additional adjustment with global WMH volume. [file 13195_2022_1149_MOESM1_ESM.docx]

**Supplementary Table 1.** Interaction effect of global WMH volume and global Aβ on inferior tau deposition

|  | β (SE) | *p*-value |
| --- | --- | --- |
| IT tau ~ WMH × global Aβ + WMH + global Aβ + age + sex + APOE4 + VRS^a^ | | |
| WMH × global Aβ | 0.003 (0.006) | 0.588 |

^a^ Summary of generalized linear regression model using global WMH volume and global Aβ deposition as interactive predictors of tau deposition.
Abbreviations: WMH, white matter hyperintensity; Aβ, β-amyloid; APOE4, apolipoprotein E ε4 positivity; VRS, vascular risk score; SE, standard error.

**Supplementary Table 2.** Interaction effect of BP marker and global Aβ on inferior temporal tau deposition with additional adjustment with global WMH volume

|  | β (SE) | *p*-value |
| --- | --- | --- |
| IT tau ~ BP marker × global Aβ + BP marker + global Aβ + age + sex + APOE4 + WMH + VRS^a^ | | |
| History of hypertension × global Aβ | 0.190 (0.153) | 0.220 |
| Current SBP (continuous) × global Aβ | 0.011 (0.004) | 0.006 |
| Current DBP (continuous) × global Aβ | 0.019 (0.006) | 0.002 |

^a^ Summary of generalized linear regression model, repeated for each blood pressure marker (e.g., history of hypertension or current blood pressure level) and global Aβ as interactive predictors of tau deposition.
Abbreviations: BP, blood pressure; Aβ, β-amyloid; WMH, white matter hyperintensity; APOE4, apolipoprotein E ε4 positivity; VRS, vascular risk score; SBP, systolic blood pressure; DBP, diastolic blood pressure; SE, standard error.
